# Assessing the role of anastomotic level in low anterior resection (LAR) surgery among rectal cancer patients in the development of LAR syndrome: a systematic review study

**DOI:** 10.1186/s12893-023-02166-5

**Published:** 2023-09-01

**Authors:** Mohammad Reza Hashempour, Muhammadhosein Moradi, Reza Ghasemian oroomi, Siamak Daneshvar, Alipasha Meysamie, Mohammadreza Nikshoar, Fakhrosadat Anaraki

**Affiliations:** 1 Colorectal Division of Surgical Ward, Taleghani Hospital, Shahid Beheshti University of Medical Sciences, Tehran, Iran; 2 School of Medicine, Shahid Beheshti University of Medical Sciences, Tehran, Iran; 3 Community and Preventive Medicine Department, Medical Faculty, Tehran University of Medical Sciences, Tehran, Iran

**Keywords:** Low anterior resection syndrome, Rectal neoplasms, Anastomosis, Postoperative complications

## Abstract

**Background:**

The etiology of LARS has not been elaborated on clearly. Studies have reported neoadjuvant therapy, low-lying rectal cancers, adjuvant therapy and anastomotic leakage as risk factors for the development of LARS. Anastomotic level has also been proposed as a possible risk factor; However, there have been conflicting results. This study aims to evaluate the role of the level of anastomosis as a potential risk factor for the development of LARS.

**Method:**

A systematic literature search was conducted on Pubmed, Scopus, Embase, and Web of Science databases using Mesh terms and non-Mesh terms from 2012 to 2023. Original English studies conducted on rectal cancer patients reporting of anastomotic level and LARS status were included in this study. Eligible studies were assessed regarding quality control with Joanna-Briggs Institute (JBI) questionnaires.

**Results:**

A total of 396 articles were found using the research queries, and after applying selection criteria 4 articles were selected. A sample population of 808 patients were included in this study with a mean age of 61.51 years with male patients consisting 59.28% of the cases. The Mean assessment time was 15.6 months which revealed a mean prevalence of 48.89% for LAR syndrome. Regression analysis revealed significantly increased risk of LAR syndrome development due to low anastomosis level in all 4 studies with odds ratios of 5.336 (95% CI:3.197–8.907), 3.76 (95% CI: 1.34–10.61), 1.145 (95% CI: 1.141–2.149) and 2.11 (95% CI: 1.05–4.27) for low anastomoses and 4.34 (95% CI: 1.05–18.04) for ultralow anastomoses.

**Conclusions:**

LARS is a long-term complication following surgery, leading to reduced quality of life. Low anastomosis level has been reported as a possible risk factor. All of the studies in this systematic review were associated with an increased risk of LARS development among patients with low anastomosis.

## Introduction

Colorectal cancers (CRCs) are the fourth most prevalent malignancies worldwide and stand as the second leading cause of cancer-related mortality, with 7.9% of all new cancer cases and accounting for 8.6% of all cancer-related deaths [1, 2], with one-third of cases originating in the rectum [3]. To achieve better oncologic outcomes, total mesorectal surgery (TME), a sphincter-saving surgery (SPS), was introduced and superseded the conventional abdominoperineal resection (APR) for mid- and low-level rectal cancers and led to significantly better local recurrence control alongside improvements in adjuvant and neoadjuvant therapies [4–6]. However, despite these strides in cancer management, many post-operative complications have been observed, with impaired bowel function as the most common complication [7, 8]. Rectal cancer survivors experience various symptoms, including incontinence to flatus/feces, urgency, increased defecation frequency, unpredictable bowel movements, and emptying difficulty following surgical therapy, collectively called low anterior resection syndrome (LARS) [9, 10]. LARS significantly influences the patient's life, lowers the quality of life, and imposes social constraints for up to 15 years [11–13]. In some studies, the prevalence of LARS varies from 30% up to 80%-90%. Improvements have been reported up to 18 months following the surgery [7, 9]; however, 50% of the cases are reported to have standing, long-term complications [14].

The etiology of LARS has yet to be elaborated on clearly. Anatomic and physiological factors are intricately involved in bowel function alteration. Low anterior resection (LAR) surgery can disrupt the anatomical integrity and physiologic homeostasis by inflicting damage to the anorectal nervus plexus, damage to the anal sphincter, and reduction in the anal reservoir [6]. Studies have reported neoadjuvant therapy, low-lying rectal cancers, adjuvant therapy [15, 16], and anastomotic leakage [17, 18] as risk factors for the development of LARS.

Anastomotic level, or anastomotic height, has emerged as a possible risk factor, as low-lying tumors and anastomoses amplify the risk of compromising the anal sphincter and candidate the patient to undergo neoadjuvant radiotherapy. However, there have been conflicting results regarding the independent role of anastomotic height [19]. As a result, the identifying low-height anastomoses as a possible independent risk factor is important and may propose alterations in the surgical techniques. Due to the prevalence, long-term character of this complication, and its major personal, social, and emotional effect on the patients' life, this study aims at assessing the role of the level of anastomosis as a possible risk factor on the development of LARS.

## Methods

The current study was performed according to the Preferred Reporting Items for Systematic Reviews and Meta-Analyses (PRISMA) 2020 checklist to assess the role of anastomotic height in the occurrence of LARS or anastomotic leakage in rectal cancer survivors.

### Literature search

A systematic literature search was conducted on Pubmed, Scopus, Embase, and Web of Science databases using “low anterior resection syndrome”, “anterior resection syndrome”, “LARS”, “LAR syndrome”, “low anterior resection syndrome score”, “anastomotic level”, “anastomotic height”, “anastomosis level”, and “anastomosis height” using “AND” and “OR” Boolean operators from 2012 to 2023. The acquired articles were initially screened by title and abstract to assess their relevance. Selected studies were then evaluated based on the full-text analysis and relevance. Furthermore, chosen studies’ reference lists were screened as well.

### Study selection

#### Inclusion criteria

Among the search results, original studies such as clinical trials, cohort studies, and cross-sectional descriptive studies with the evaluation of anastomotic level from the anal verge, and assessment of the LARS (preferably LARS score [20]), within at least 6 months from the operation were included.

#### Exclusion criteria

Review, and case-report studies were excluded. Studies with LAR surgery for reasons other than rectal cancer were excluded. Tumor location or tumor level was not used as a substitute. Non- English articles were excluded as well.

#### Quality assessment

**Fig. 1:**
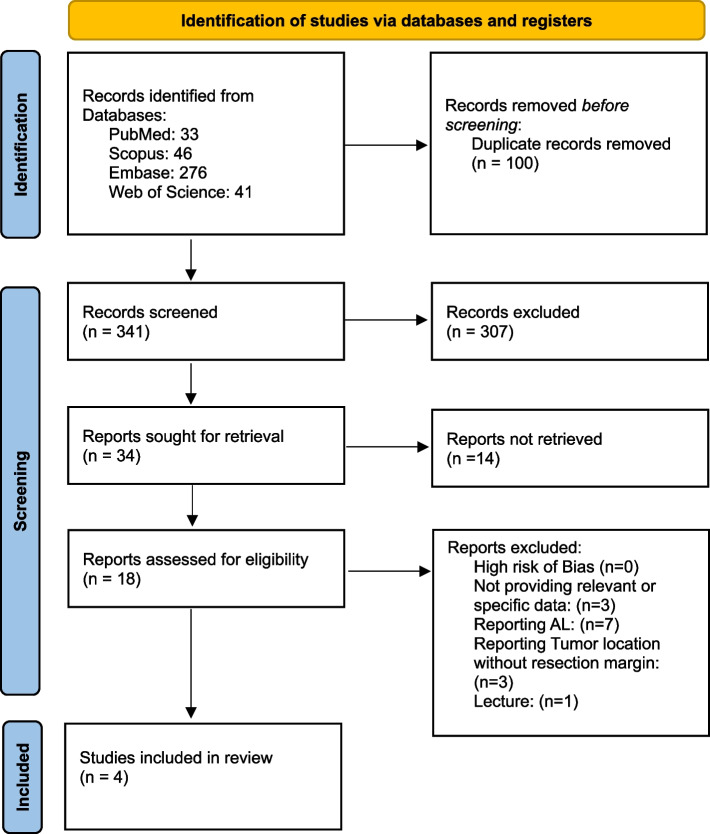
Flowchart diagram of database search and inclusion of the studies; AL: Anastomosis leak

Studies that were selected in the screening process, were then assessed for quality based on Joanna Briggs institute (JBI) tools regarding their study type. The quality control process was performed independently by two different authors. Figure 1 presents a flowchart diagram of the search and inclusion process.

#### Data extraction

Data regarding patients demographics (age, and sex), their LARS status, and level of anastomosis was recorded. Also, univariate and multivariate analysis findings assessing the odds of development of LARS in the setting of low-level anastomosis were documented as well.

## Results

A total of 396 articles were found using the research queries. Following the exclusion of duplicate studies, 341 papers have then entered the title and abstract screening process. Eighty-five articles were selected following the title and abstract screening and following full-text assessment, a total of 34 studies were included, 18 of which could be accessed, among whom only 4 articles were selected.

**Fig. 2:**
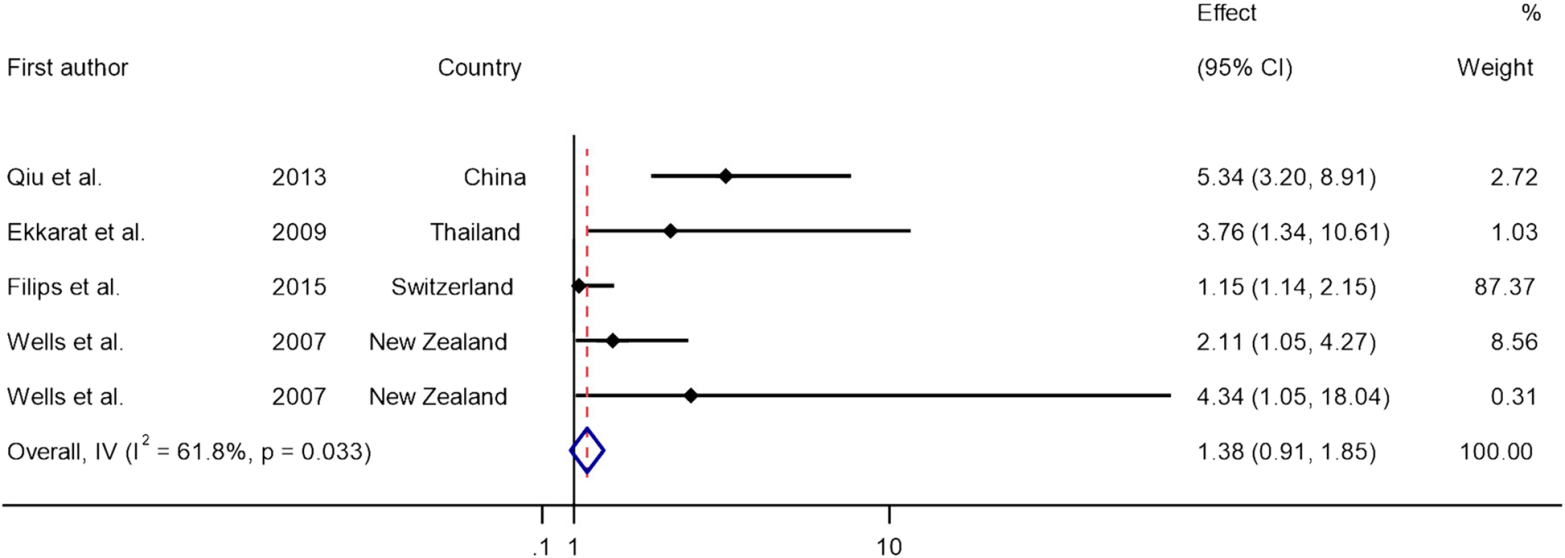
Forest plot of the included studies assessing the homogeneity

Four articles consisting of 2 case series, 1 cohort and 1 cross-sectional descriptive study and a sample population of 808 patients conducted in China, Thailand, Switzerland, and New Zealand were evaluated. Two of the studies provided quantitative data on the anastomosis level, presenting it as the mean distance measured from the anal verge, while two studies presented qualitative observations of the anastomosis level. In these cases, the assessment involved categorizing the anastomosis as being either proximal or distal to a defined threshold of 5 cm from the anal verge. Studies evaluated LARS status within a mean of 15.6 months after surgery with two studies assessing the patients after 12 months, 1 study after 6 months and 1 study after 18 months. The mean age of the patients was 61.51 years with a predominance of 59.28% male patients. Homogeneity analysis was performed using forest plot yielding *I*^2^ = 61.8% with *p* = 0.033 which suggests high heterogeneity among studies. Figure 2 demonstrates the forest plot. A summary of the included studies is provided in Table 1.

**Table 1:** Summary of the studies

**ID**	**Authors**	**Country**	**Year**	**Population**	**Age**	**Sex**		**Anastomosis height (cm)**	**No LARS**	Minor/ Major LARS
						**Male**	**Female**			
**1**	Yuan Qiu et al	China	2013	337	61.03	208 (61.72)	337	LA: 131 (38.87) HA: 206 (61.12)	211 (62.61)	126 (37.39)
**2**	Patomphon Ekkarat et al	Thailand	2009	129	60.2	67 (51.94)	129	7.6	84 (65.12)	45 (34.88)
**3**	Alexandra Filips et al	Switzerland	2015	80	62.0	53 (66.25)	80	4	26 (32.5)	64 (80)
**4**	Wells et al	New Zealand	2017	262	68.8	151 (57.63)	262	LA: 131 (50) HA: 131 (50)	102 (38.93)	160 (61.07)

LARS score is a questionnaire consisting of 5 questions, ranging from 0 to 42, with 0 to 20, 21 to 29, and 30 to 42, regarded as No LARS, Minor LARS, and Major LARS, respectively [21]

Qualitative data are presented as count (percent); Quantitative data are presented as mean

*LA*: Low anastomosis (< 5cm from the anal verge), *HA* High anastomosis (> 5cm from the anal verge)

The prevalence of LARS ranged from 34.88% to 80%, with a mean prevalence of 48.89% among the total 808 patients. The frequency of no LARS cases following surgery was 62.61%, 65.12%, 32.5%, and 38.93% in Yuan Qiu et al. [7], Ekkarat et al. [4], Filips et al. [14], and Wells et al. [11] studies, after 18, 12, 6, and 12 months, respectively, with the lowest frequency of no LARS cases in Filips et al. [14] and the highest in Yuan Qiu et al. [7] study. Major LARS was reported in Yuan Qiu et al. [7], Ekkarat et al. [4] and Filips et al. [14] studies with a prevalence of 18.69%, 17.83%, and 36.25%, respectively. A summary of LARS subgroups is provided in Table 2.

**Table 2:** LARS prevalence distribution based on severity of the cases

**ID**	**Authors**	**Population**		**LARS**	
			**No**	**Minor**	**Major**
**1**	Yuan Qiu et al	337	211 (62.61%)	63 (18.69%)	63 (18.69%)
**2**	Patomphon Ekkarat et al	129	84 (65.12%)	22 (17.05%)	23 (17.83%)
**3**	Alexandra Filips et al	80	26 (32.5%)	35 (43.75%)	29 (36.25%)
**4**	Wells et al	262	102 (38.93)	*	*

^*^Minor and Major LARS cases are not provided separately

Anastomosis level was mentioned in two studies as high and low anastomosis with 38.87% anastomoses within 5 cm of the anal verge and 61.12% high anastomoses upper than 5 cm from anal verge in Yuan Qiu et al. study [7], and 50% high anastomosis (anastomosis to intraperitoneal rectum), 36.25% low (anastomosis to extraperitoneal rectum), and 13.74% ultralow (coloanal anastomosis) in Wells et al. study [11]. Quantitative reports presenting the mean ± SD of the distance of anastomosis from the anal verge were provided by Ekkarat et al. [4] and Filips et al. [14] with 7.6 ± 3.5 cm (mean ± SD), and 4 ± 1.48 cm, respectively.

In three studies, minor and major LARS has been defined addressed separately which is presented in Table 2. Yuan Qiu et al. [7] reported an odds ratio (OR) of 5.336 (95% CI:3.197–8.907) of low anastomosis for the development of LARS syndrome with univariate analysis. Ekkarat et al. [4] reported an OR of 3.76 (95% CI: 1.34–10.61), and Filips et al. [14] reported 1.145 (95% CI: 1.141–2.149). In the Wells et al. [11] study, using logistic regression analysis, an OR of 2.11 (95% CI: 1.05–4.27) for low anastomoses and 4.34 (95% CI: 1.05–18.04) for ultralow anastomoses were reported at 12 months after surgery. A summary of OR of the studies with 95% CI is provided in Table 3.

**Table 3:** Summary of odds ratio regarding low anastomosis level and development of LARS

ID	Authors	OR	95% CI
**1**	Yuan Qiu et al	5.336	3.197–8.907
**2**	Patomphon Ekkarat et al	3.76	1.34–10.61
**3**	Alexandra Filips et al	1.145	1.141–2.149
**4**	Wells et al.^a^ (low)	2.11	1.05–4.27
**5**	Wells et al.^b^ (Ultralow)	4.34	1.05–18.04

^a^Low anastomosis

^b^Ultralow anastomosis

## Discussion

LARS encompasses a complex set of symptoms and complications following sphincter preserving surgeries (SPS), anterior resection, and low anterior resection, with good local recurrence control, yet, poor functional outcomes such as fecal urgency, fecal incontinence, and increased bowel movements [21]. Although the use of TME instead of APR yielded poor functional despite better oncologic outcomes; however, no significant difference has been observed among surgical approaches such as laparotomy, laparoscopy, and robotic-assisted approach in the TME technique for LARS development [7, 11, 22]. Several questionnaires have been developed to assess the severity of the patient’s symptoms, such as the Wexner questionnaire and LARS score [20], which are currently the most widely used assessment tools [14]. Different mechanisms have been proposed as the underlying pathophysiologies of the disease. Primarily, disruption of the mucosal integrity was proposed; however, long-lasting complications and the persistence of symptoms beyond two years following the surgery [11] suggested other mechanisms, such as anal sphincter impairment due to iatrogenic damage causing decreased mean resting anal pressure, translating into patients’ reduced continence [4], decreased neorectum capacity and innervation [12], and abnormal gastrointestinal motility as a result of surgical manipulations [19].

Regarding its unclear etiology and as a preventative attempt, recent endeavors have sought to unravel the risk factors for developing LARS due to its prolonged nature and lack of definitive therapy. Several factors have been reported as potential risk factors, among which adjuvant therapy [23] has been the most extensively assessed. Despite the evaluation of surgical techniques [7], tumor location [24], anastomosis complications, and anastomosis techniques [12], anastomotic height has yet to be studied as much.

While evaluating the level of anastomosis and the risk of LARS development, Ekkarat et al. suggested a 5 cm cut-off for anastomosis height [4]. Surgical manipulation in low-lying rectal cancers is usually more extensive and increases the risk of nerve damage to the pudendal nerve and the rectal wall nerve plexus [25]. Similarly, lower tumors and lower anastomoses increase the risk of anal sphincter injury, especially in male patients with less pelvic diameter; however, the role of sex is still under investigation [13]. As the anastomosis level draws nearer to the anal verge, the rectal remanent decreases, and the need to form the neorectum becomes prominent. Neorectum has less compliance than the rectum, which can be attributed to less neorectum wall thickness and innervation [19]. A higher prevalence of anastomotic complications, including higher anastomotic leakages, has been reported in low anastomoses [22, 26], in which perfusion impairment due to injury to small arteries has been proposed as the underlying cause [18]. However, presenting the level of anastomosis as an independent risk factor for developing LARS needs further consideration. Low tumor level has been proposed as a risk factor for developing LARS, and anastomosis level is closely tied to the tumor level. Moreover, the selection of different surgical approaches with varying complications is also based on the tumor level. Thus, defining the role of anastomotic height as an independent risk factor or as an indicator of other risk factors’ concurrence is important.

Despite highlighting the knowledge gap regarding the role of anastomosis height in LARS, this review has limitations. Two of the included studies were case series, and all of these studies evaluated for LARS in brief follow-up periods of 6, 12, and 18 months, while LARS is considered a long-term complication. Moreover, despite our efforts, we could not access the data needed for a meta-analysis.

In conclusion, LARS is a long-term complication following surgery, leading to reduced quality of life. Low anastomosis level has been reported as a possible risk factor, and all of the studies in this systematic review were associated with an increased risk of LARS development among patients with low anastomosis. The lack of evidence in this field emphasizes the need for more comprehensive studies to achieve a more definitive conclusion.

## Data Availability

Data are available upon request from the corresponding author.

## Abbreviations

CRC: Colorectal cancer
TMS: Total mesorectal surgery
APR: Abdominoperineal resection
LARS: Low anterior resection syndrome
LAR: Low anterior resection
PRISMA: Preferred reporting items for systematic reviews and meta-analyses
JBI: Joanna Briggs institute
